# Supplementary material for: Hesperidin Reverses Oxidative Stress-Induced Damage in Kidney Cells by Modulating Antioxidant, Longevity, and Senescence-Related Genes
Source: Biomedicines. 2025 Dec 9;13(12):3016. doi: 10.3390/biomedicines13123016 (PMC12730979; doi:10.3390/biomedicines13123016)
Supplement: Supplementary file 1 [file biomedicines-13-03016-s001.zip › Supplementary Figure S1 and S2.pdf]

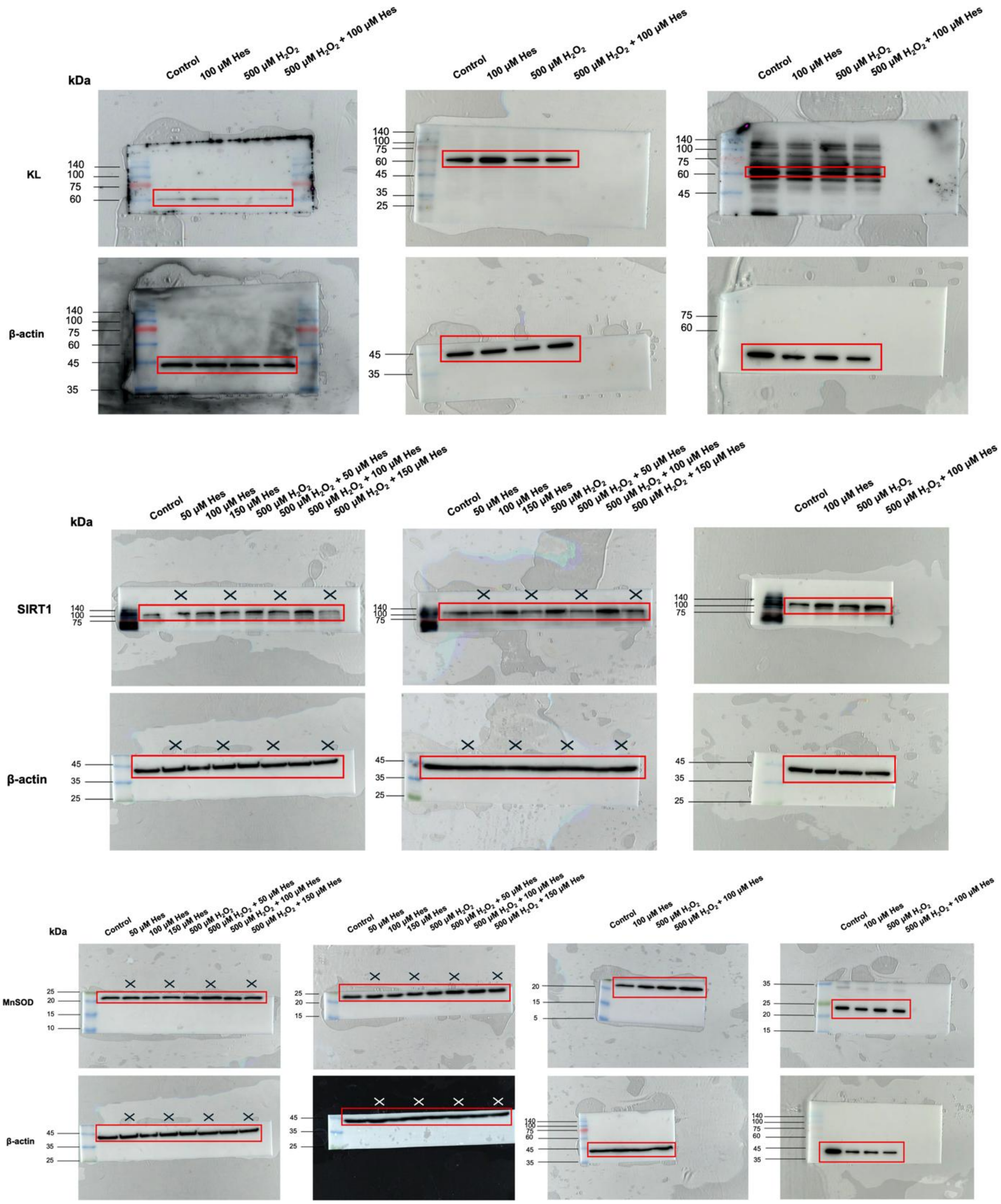

Figure S1. Uncropped western blot images of KL, SIRT1 from three independent experiments, and MnSOD from four independent experiments.

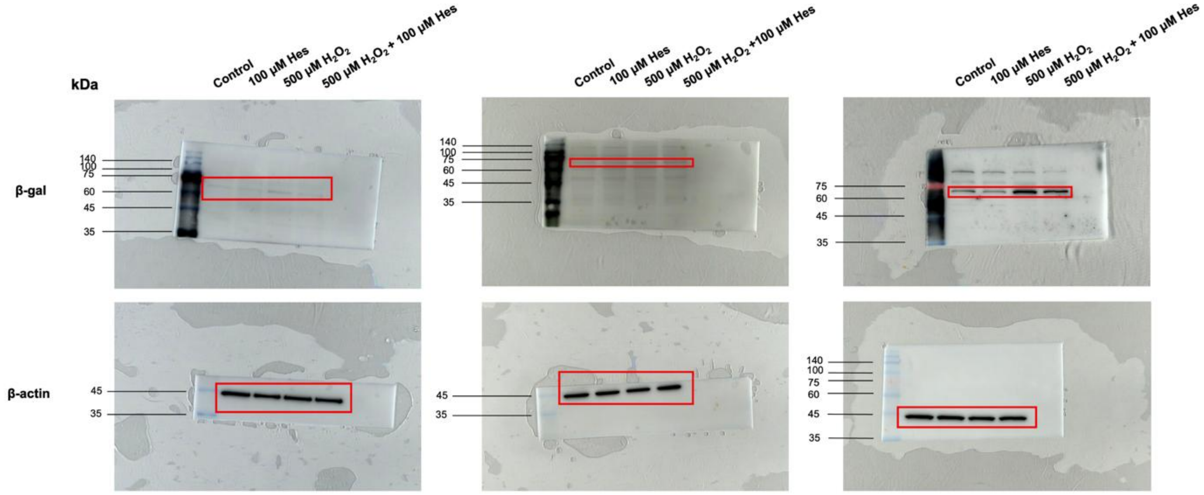

Figure S2. Uncropped western blot images of β-galactosidase from three independent experiments.
